# Combining Behavioral and ERP Methodologies to Investigate the Differences Between McGurk Effects Demonstrated by Cantonese and Mandarin Speakers

**DOI:** 10.3389/fnhum.2018.00181

**Published:** 2018-05-04

**Authors:** Juan Zhang, Yaxuan Meng, Catherine McBride, Xitao Fan, Zhen Yuan

**Affiliations:** ^1^Faculty of Education, University of Macau, Macau, China; ^2^Department of Psychology, The Chinese University of Hong Kong, Shatin, Hong Kong; ^3^School of Humanities and Social Science, The Chinese University of Hong Kong, Shenzhen, Shenzhen, China; ^4^Faculty of Health Sciences, University of Macau, Macau, China

**Keywords:** audiovisual speech perception, Cantonese, Mandarin, McGurk effect, mismatch negativity

## Abstract

The present study investigated the impact of Chinese dialects on McGurk effect using behavioral and event-related potential (ERP) methodologies. Specifically, intra-language comparison of McGurk effect was conducted between Mandarin and Cantonese speakers. The behavioral results showed that Cantonese speakers exhibited a stronger McGurk effect in audiovisual speech perception compared to Mandarin speakers, although both groups performed equally in the auditory and visual conditions. ERP results revealed that Cantonese speakers were more sensitive to visual cues than Mandarin speakers, though this was not the case for the auditory cues. Taken together, the current findings suggest that the McGurk effect generated by Chinese speakers is mainly influenced by segmental phonology during audiovisual speech integration.

## Introduction

Speech perception has been extensively explored as a unimodal process of perceiving and distinguishing auditory information (Obleser et al., 2006; Zhang and McBride-Chang, 2010). However, the nature of speech perception is actually bimodal, involving both the auditory and the visual modalities, particularly in the case of face-to-face communication (Munhall et al., 2004). Visual information can be perceived from the movements of the lips (tongue), which can be utilized to judge whether sounds are lip-articulated (labials) or non-lip-articulated (non-labials) (Sekiyama, 1997; Chen, 2001). Visual information can also be supplementary to the perception of auditory cues in noisy environments or in unfamiliar language settings (Sumby and Pollack, 1954). What’s more, visual cues can remarkably modify auditory perception even when the auditory source is clear. A dramatic demonstration of this phenomenon is the McGurk effect.

The McGurk effect refers to the illusory perception of audiovisual speech when the auditory and visual cues are incongruent (McGurk and MacDonald, 1976). For instance, when dubbing auditory /pa/ onto visual /na/, participants may report the perception of /ta/, of which the place of articulation is consistent with visual /na/ whereas the manner of articulation is inherited from the auditory /pa/. More interestingly, the illusion is persistent even when perceivers have been aware of the existence of the effect (Macdonald and McGurk, 1978).

Many studies have been carried out to explore the integration mechanism of the McGurk effect and factors that may influence the effect. For example, some inter-linguistic comparison studies investigated the McGurk effect exhibited by Chinese speakers and those speaking other languages in order to understand the utilization of visual cues in McGurk illusion across languages (Sekiyama, 1997; Hayashi and Sekiyama, 1998). Sekiyama (1997) assessed and compared the magnitudes of the McGurk effect among Chinese and Japanese speakers and found that Chinese speakers exhibited a weaker McGurk effect than their Japanese participants. The authors attributed this finding to the tonal characteristic of the Chinese language. Specifically, Chinese is a tonal language, and lexical tones are used by Chinese speakers to distinguish word meanings. Auditory cues, rather than visual cues, are more powerful in recognizing lexical tones in Chinese. Consequently, Chinese speakers might rely more on auditory information when perceiving inconsistent stimuli and manifest weaker McGurk effects than their Japanese counterparts. Although the finding is intriguing, this pioneering work has several limitations. The main concern of the study is the choice of the participants: In the study, Chinese participants who had lived in Japan for several years were recruited from that country. In other words, they were Chinese-Japanese bilinguals instead of monolingual Chinese speakers. More importantly, a positive correlation was identified between the magnitude of the McGurk effect demonstrated by these Chinese speakers and the years that they had lived in Japan (Sekiyama, 1997). However, for the Japanese participants, none of them had reported any experience of living abroad. Therefore, the findings by Sekiyama (1997) might result from the bilingual experience instead of from the Chinese language *per se*. Second, the results might also be due to the confounding effect of cultural difference between the two countries that the degree of face avoidance might be different during face-to-face communication. Finally, Sekiyama (1997) explained the weaker McGurk effect exhibited by Chinese speakers from the perspective of tonal characteristics of Chinese. However, there was another possibility that the weaker McGurk effect by Chinese speakers might be due to the simpler segmental structure of Chinese compared to Japanese. Specifically, Chinese is a morpho-syllabic language, in which a character usually corresponds to a syllable and a morpheme (Shu et al., 2006). However, in Japanese, one single word is usually comprised of several morae, which are the sub-syllabic units used for Japanese phonological and orthographic representations (Tamaoka and Makioka, 2009). Therefore, Japanese is more complex than Chinese in segmental phonology. Given that visual information (e.g., lip movements, the place of articulation) provide useful cues for the identification of segmental phonology especially for consonants during audiovisual speech integration, it is possible that the stronger McGurk effect by Japanese speakers in Sekiyama’s (1997) study was the result of greater utilization of visual cues when perceiving segmental phonology. Due to these three reasons, the pioneering work by Sekiyama (1997) requires additional follow-up research in order to fully reveal the role of native language background on McGurk effect with the possible confounding effects (i.e., bilingual experience, culture) eliminated.

In the present study, a systematic investigation was performed to explore the impact of tonal and segmental features of language background on the utilization of visual cues in audiovisual speech perception among representative samples from native Chinese speakers. Specifically, in the current study, Cantonese and Mandarin speakers were selected as intra-cultural participants with similar linguistic experience. The Chinese language includes different dialects such as Cantonese and Mandarin, which are different in both tonal and segmental aspects of phonology. Lexical tones in Cantonese and Mandarin are realized by the fundamental frequency (F0). Specifically, two components of F0, including the height and the direction, are essential in tone perception (Gandour, 1983; Fu et al., 1998). The height of tones refers to the average height of the pitch contour, while the direction of tones denotes the leading direction of the pitch movement within the syllables (Gandour, 1983). In Mandarin, all four tones are contour tones, differing in direction (Hume and Johnson, 2001). By contrast, there are six tones in Cantonese, including both contour and level tones (Khouw and Ciocca, 2007). Different from contour tones, the level tones share similar flat pitch contour and differ only in the relative pitch height (Lee et al., 1995; Khouw and Ciocca, 2007). To discriminate level tones, Cantonese speakers need to perceive the relative pitch differences between these tones (Bauer and Benedict, 1997; Lee and Qian, 2007). Rigid head movements may serve as supplementary cues for the perception of contour tones (Chen and Massaro, 2008; Burnham et al., 2015), but not for level tones because only minimal pitch movement is involved in level tones (Burnham et al., 2001). That is, Cantonese has a more complex tonal system and it is aurally more ambiguous than Mandarin. More importantly, visual information may be less effective in the perception of lexical tones in Cantonese than in Mandarin due to the existence of level tones.

Besides the tonal/suprasegmental aspect, the segmental phonology of Cantonese is also more complex than that of Mandarin. Cantonese has much richer phoneme and base syllable inventories than Mandarin (Lee et al., 2002). The base syllable, which refers to monosyllables without any tonal information, is about 50% more in Cantonese than in Mandarin (Lee et al., 2002). Meanwhile, plosive stops exist in Cantonese syllables (e.g., /p/, /t/, /k/), which are voiceless and difficult to be perceived auditorily (Chan and Li, 2000). Compared to Mandarin, the larger segmental inventory and the existence of plosive stops lead to the higher ambiguity in perceiving Cantonese syllables. Previous research showed that Cantonese speakers responded less accurately in recognizing auditory syllables than their Mandarin counterparts (Gao et al., 2000). Different from tonal information, the visual cues for segmental phonology can be obtained from the lip movements or the place of articulation rather than rigid head movement (Lansing and McConkie, 1999; Burnham et al., 2006). Furthermore, more visual cues can be utilized to perceive segmental information in Cantonese than in Mandarin. Statistically, there is a larger proportion of visually identified labials in Cantonese than in Mandarin (Leung et al., 2004; Chen and Kent, 2010). In addition, Cantonese has more syllable-final nasals and plosive stops, which occur in the coda positions, and more visual information is required in the processing of these codas (Chan and Li, 2000; Gao et al., 2000). Therefore, visual cues are more effective and essential for the perception of segmental phonology in Cantonese than in Mandarin.

Taken together, compared with Mandarin, Cantonese is more complex and ambiguous to distinguish aurally in both the tonal and segmental aspects. However, the effectiveness of visual cues that could be supplementary to the perception of tonal and segmental information differs between the two dialects. Those differences may have impacts on the utilization of visual cues and, consequently, the McGurk effect during audiovisual speech perception.

In the current study, two well-designed experiments were conducted to investigate speech perception under auditory-only (AO), visual-only (VO), and audio-visual (AV) conditions between Mandarin and Cantonese speakers using behavioral and event-related potentials (ERPs) measurements. According to existing literature, there could be two contradictory predictions: (a) Given that the tonal system is more complex and visual cues are less effective in distinguishing tones in Cantonese than in Mandarin, Cantonese speakers might rely more on the auditory modality and utilize less visual information and consequently exhibit a weaker McGurk effect than Mandarin speakers; (b) As the segmental phonology is more complex and more visual cues can be utilized to compensate for the auditory segmental complexity in Cantonese than in Mandarin, Cantonese speakers might rely more on the visual modality and therefore show a stronger McGurk effect than their Mandarin counterparts.

Notably, among various ERP components, mismatch negativity (MMN) was the focus of Experiment 2 because it reflects the automatic processing without the involvement of consciousness (Näätänen, 2001). MMN is a negative deflection, peaking at about 100–250 ms after stimulus onset, and is typically considered as an index of pre-attentive detection of infrequent stimuli in auditory processing (Näätänen and Alho, 1995; Näätänen, 2001). MMN is generally explored using an oddball paradigm, in which standard and deviant stimuli are presented randomly with different frequencies and MMN is generated by subtracting the ERP waveforms elicited by standard stimuli from those elicited by deviant ones. MMN can be evoked by changing simple physical features of auditory sounds (Näätänen et al., 2005) or by manipulating more complex speech sources (Näätänen, 2001; Tsang et al., 2011). Previous work has revealed that the auditory MMN is generated from two brain areas (Näätänen et al., 2007; Saint-Amour et al., 2007): the supratemporal auditory cortex bilaterally and the right-hemisphere frontal cortex. Interestingly, MMN is not only found for auditory stimuli, but also exists in the visual modality. Visual MMN (vMMN) is quite similar to auditory MMN (Czigler, 2007) but it is generated in the posterior parietal-occipital and posterior occipital-temporal cortex, peaking at 100–250 ms after stimulus onset (Flynn et al., 2009; Fisher et al., 2010). Recent work has also reported that auditory MMN could be evoked in bimodal audiovisual speech perception even when the real acoustic variation was absent. The change of visual cues in audiovisual speech integration could activate the bilateral supratemporal auditory cortex (Flynn et al., 2009; Kreegipuu et al., 2013). For instance, Sams et al. (1991) dubbed auditory /pa/ onto frequent visual stimulus /pa/ and infrequent visual stimulus /ka/ using the McGurk paradigm, which resulted in the congruent audiovisual speech perception of /pa/ and incongruent illusion of /ta/, respectively. During the dubbing process, only visual cues were altered, while the auditory cues were kept consistent. The change of visual cues elicited the mismatch fields (MMFs), appearing at about 180 ms after stimulus onset in the supratemporal cortex, as recorded using a neuromagnetic technique.

## Materials and Methods

### Ethics Statement

In both experiments, written informed consent was obtained from each participant prior to the formal testing and the experimental procedures were approved by the Ethics Committee of the University of Macau. All methods were carried out in accordance with the approved guidelines and regulations.

### Experiment 1

By comparing Cantonese and Mandarin speakers, Experiment 1 was conducted to examine the relationship between the McGurk effect and dialect background in Chinese. In this study, the perceptions of audio-only, visual-only (VO), and audiovisual (AV) stimuli were compared between the two groups.

#### Participants

In total, 15 native Cantonese speakers (5 males and 10 females, mean age: 24.0 years) and 15 native Mandarin speakers (6 males and 9 females, mean age: 24.7 years) participated in this experiment. All the subjects were right-handed and had normal hearing and normal or corrected-to-normal vision. The consent of all participants was obtained prior to the testing.

#### Stimuli and Procedures

In this experiment, eight syllables, including /ba/, /pa/, /ma/, /da/, /ta/, /na/, /ga/, and /ka/ were adopted as our experimental materials (Sekiyama, 1997; Hayashi and Sekiyama, 1998). The eight syllables were further grouped into two categories: labials (/b/, /p/, /m/) and non-labials (/d/, /t/, /n/, /g/, /k/). All of the syllables were pronounced by a male native English speaker and the utterances were recorded by Canon 70D with minimal head movements to produce the visual signals of the videotapes. In addition, for our task design, the auditory stimuli were recorded in an anechoic room by Sennheiser EW 100 ENG G3 (Wireless Lapel Microphone). Then 8 AO and 8 VO stimuli were synthesized frame by frame by using the Corel VideoStudio Pro X7, and as a result, a total of 64 (8 visual by 8 audio) AV stimuli were produced.

All the materials were randomly presented using E-prime 2.0. In each trial of the experiment, participants were asked to report what they had heard and all the answers were open-ended. For example, when dubbing auditory /pa/ onto visual /na/, the participants might report the perception of /ta/ or /npa/. Three conditions, namely aural only, VO (visual only), and AV (audiovisual), were included in the experiment. In the three conditions, each stimulus was presented six times and lasted for 3200 ms with an inter-stimuli interval (ISI) of 2000 ms. In the AO condition, the auditory stimuli were presented binaurally through headphones with black frames on the computer and in the VO condition, only videos were presented without auditory sounds.

### Experiment 2

#### Participants

Twenty-four college students were recruited as participants in Experiment 2, and none of them had participated in Experiment 1. Half of the participants were native Cantonese speakers (4 males and 8 females, mean age: 23.0 years) and the other half were Mandarin speakers (5 males and 7 females, mean age: 24. 5 years). All participants had normal or corrected-to-normal vision and no hearing disorder. Each participant signed an informed consent form before the formal test.

#### Stimuli and Procedures

Three kinds of stimuli, namely auditory-only (AO), visual-only (VO), and audiovisual (AV) syllables, were used as experimental materials. The utterances were generated from a male native English speaker and were digitally recorded (frame rate: 25 images/s; audio sampling rate: 44,100 Hz in 16 bits), with both the head and the upper part of the shoulder being shot into the video. The acoustic /ba/ and /ga/ were standardized to be 360 ms long with an intensity of 65 dB SPL and the visual stimuli were edited as 720 ms long. Four sets of tokens were generated, including two unimodal visual tokens, two unimodal acoustic tokens, congruent AV token, and incongruent AV token. Congruent AV token was acoustic /ba/ dubbed on visual /ba/ and incongruent AV token was acoustic /ba/ dubbed on visual /ga/. For both congruent and incongruent AV tokens, the articulation movements started 120 ms prior to the onset of the audio stimuli. The incongruent AV token elicited a McGurk effect such that almost all of the participants reported a clear perception of /da/ after participating in this experiment. All the visual stimuli were presented in the center of a 19-in color monitor located 80cm in front of the participant and the auditory stimuli were presented binaurally through headphones. During the experiment, participants were required to rest their chins on a holder to avoid head movements.

The oddball paradigm was adopted and three conditions (AO, VO, and AV) were presented separately. There were four blocks for each condition and the average duration for each block was kept within 5 min to minimize the habituation effect resulted from the long exposure to the stimuli. In order to control the learning effect that may be generated through discriminating visual stimuli in the AV condition, AV and AO conditions were presented first in a counterbalanced sequence among participants (AV-AO or AO-AV). In each trial, a 500 ms fixation was presented, followed by the stimulus and then a 500 ms blank. In each block, 10 standard trials were presented first, followed by another 150 trials including 20% deviants and 80% standard trials. The presentation of these trials in each block was pseudorandomized. Participants could have a short break between every two blocks.

#### Recording and Analysis

The experiment was presented using E-Prime 2.0 which was linked to Net Station 4.5.6 software. Continuous EEG was recorded from the EGI 128 electrode Hydro Cel Geodesic Sensor Net with a sampling rate of 1000 Hz in a sound attenuated experimental lab. During the recording, the impedances for all electrodes were kept below 50 kΩ before each block. The data were processed with EGI Net Station Waveform Tools off-line and filtered with a 0.1–30 Hz band-pass. Then the EEG data were segmented into 1100 ms epochs (-180–920 ms surrounding the onset of auditory stimulation). The first 10 standard trials and the standard trials presented immediately after deviants in each block were excluded for further analysis. An artifact detection criterion of ± 27.5 μV was adopted to detect eye movement and ± 70 μV was used to detect eye blink for all scalp sites. The segment was considered as invalid if the difference between the maximum and the minimum amplitudes was greater than 200 μV. Meanwhile, channels were considered as bad in recording if more than 20% of the segments were invalid. Additionally, segments with more than 10 bad channels were discarded. Bad channels were replaced using the average value of surrounding channels and the ERP segments were then averaged for each participant. According to previous investigations on different reference techniques (Yao, 2001), all waveforms were referenced to the average of all 128 electrodes, and a 100 ms baseline correction was applied.

The analysis windows were based on a visual inspection of the grand average waveform and checked against data for each individual participant. The mean amplitudes were calculated for electrodes Fz, FP1/FP2, Pz, P3/P4, P7/P8, T7/T8, PO3/PO4, PO7/PO8, O1/O2. Repeated measure analyses of variance (ANOVAs) were conducted with groups (Cantonese speaker vs. Mandarin speaker) being the between-subject factor and electrode being the within-subject factor. Statistical significance was corrected through a Greenhouse-Geisser adjustment at the level of 0.05.

## Results

### Experiment 1

Following previous studies (Sekiyama, 1997), the responses given by participants were considered as correct if they fell into the same phonological category (labials or non-labials) of the auditory stimuli presented. Otherwise, the responses were treated as wrong. For example, the responses to auditory non-labials (/d/, /t/, /n/, /g/, /k/) were considered as correct if the responses of participants were still non-labials. In contrast, if the responses given by participants were labials, they would be treated as wrong. The mean accuracy and standard deviation for the AO and VO conditions and the pure McGurk effect for the AV condition are shown in **Table 1**.

**Table 1:** The mean accuracy and standard deviation for the audio-only and visual-only (VO) conditions and those for the pure McGurk effect in the AV condition for the two groups.

	Mandarin		Cantonese	
		
	*M*	*SD*	*M*	*SD*
Auditory labials	0.40	0.11	0.51	0.22
Auditory non-labials	0.99	0.01	0.97	0.05
Visual labials	0.99	0.01	1.00	0.00
Visual non-labials	0.99	0.03	0.99	0.02
McGurk effect (auditory labials with visual non-labials)	0.45	0.15	0.60	0.24
McGurk effect (auditory non-labials with visual labials)	0.35	0.23	0.46	0.26


#### AO Condition

To investigate the group difference in the aural only condition, repeated two-way ANOVAs were performed with stimulus types (labials vs. non-labials) as the within-subject factor and groups (Mandarin group vs. Cantonese group) as the between-subject factor. The main effect of stimuli was significant, *F*(1,28) = 247.38, *p* < 0.05, η^2^ = 0.89. However, the main effect of groups failed to approach significance, *F*(1,28) = 2.04, *p* = 0.16. Meanwhile, the interaction between stimuli and groups was non-significant, *F*(1,28) = 3.88, *p* = 0.07.

#### VO Condition

Similarly, repeated 2 (stimuli: labials vs. non-labials) × 2 (groups: Mandarin speakers vs. Cantonese speakers) ANOVAs were conducted for the VO condition. Only the main effect of stimuli approached significance, *F*(1,28) = 7.26, *p* < 0.05, η^2^ = 0.21. The main effect of group was non-significant, *F*(1,28) = 0.04, *p* = 0.85. Meanwhile, the interactions between stimuli and group was also non-significant, *F*(1,28) = 2.78, *p* = 0.11. To further compare the performance of the two groups, *t-*tests were conducted for labials and non-labials, respectively. Non-significant group differences were found for labials, *t*(28) = -1.00, *p* = 0.33 and for non-labials, *t*(28) = 0.71, *p* = 0.48.

#### AV Condition

Regarding the magnitude of the McGurk effect, previous work suggested that errors that participants made in the McGurk paradigm might be partly due to pure auditory perception (Sekiyama, 1997; Hayashi and Sekiyama, 1998). For example, if the participant incorrectly perceives /k/ as /p/ in a pure auditory task 15% of the time and makes the same perception errors in the McGurk condition 45% of the time, then the magnitude of the pure McGurk effect should be 30%, which can be obtained by subtracting the error rate in the pure auditory task from that made in the audiovisual task. In Experiment 1, we adopted the same means to calculate the pure McGurk effect, which was shown in **Table 1**.

To compare the pure McGurk effect between the two groups (Mandarin group vs. Cantonese group), repeated two-way ANOVAs were performed with stimulus types as the within-subject factor and groups as the between-subject factor. The main effect of stimulus types was significant, *F*(1,28) = 5.40, *p* < 0.05, η^2^ = 0.16. Specifically, dubbing auditory labials on visual non-labials elicited a stronger McGurk effect than dubbing auditory non-labials on visual labials. More importantly, the main effect of language was also significant, with Cantonese speakers exhibiting a larger McGurk effect than Mandarin speakers, *F*(1,28) = 4.61, *p* < 0.05, η^2^ = 0.14. However, the interaction of the two factors was not significant, *F*(1,28) = 0.11, *p* = 0.74.

In summary, we found that Cantonese speakers exhibited a stronger McGurk effect than Mandarin speakers in Experiment 1. This finding might be due to the lower reliance on auditory cues by Cantonese speakers, their greater utilization on visual cues, or both. Perhaps both reasons factored into this finding. Owing to the low sensitivity of behavioral measurements in quantifying the possible factors that might result in the different McGurk effects between the two groups in Experiment 1, electrophysiological (EEG) recordings were performed and analyzed in Experiment 2 to reveal the complex neural mechanisms underlying language background and the McGurk effect (Luck, 2014). If the group difference was identified for certain conditions [e.g., visual only (VO), aural only, or audiovisual (AV)] using ERPs, the reason for the group discrepancy as revealed by behavioral data in Experiment 1 could be more clearly understood.

### Experiment 2

**FIGURE 1:**
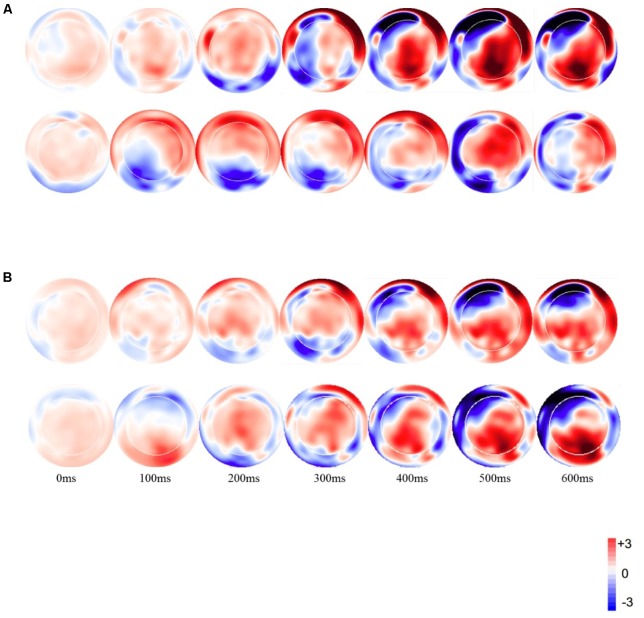
Topographic distribution of the mean amplitude in the time windows from 0 to 600 ms for the deviant minus standard difference for **(A)** visual stimuli, **(B)** audiovisual stimuli (the Upper row for each condition is for Mandarin speakers).

**Figure 1** displays the topographic voltage maps by subtracting the standard from the deviants for the VO and AV conditions.

#### AO Condition

**FIGURE 2:**
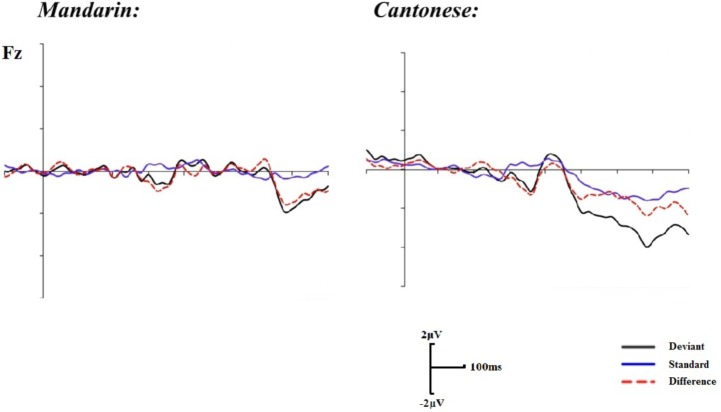
Grand mean event-related potentials (ERPs) for the standard, deviant trials, and difference waveforms in the auditory condition.

Visual inspection of the grand average of ERPs suggested that a typical MMN waveform existed in the time window of 130–360 ms after auditory stimuli onset (**Figure 2**) and the mean amplitude of the difference wave was used in analyses. A 2 (groups: Mandarin group vs. Cantonese group) × 16 (electrodes: Fz, FP1/FP2, Pz, P3/P4, P7/P8, T7/T8, PO3/PO4, PO7/PO8, O1/O2) repeated ANOVAs was implemented. Statistical analyses revealed that there was no significant main effect of group, *F*(1,22) = 1.60, *p* = 0.22. However, the main effect of electrodes approached significance, *F*(15,330) = 3.85, *p <* 0.01, η^2^ = 0.15. In addition, no significant interaction was revealed, *F*(15,330) = 0.55, *p* = 0.69. Furthermore, *post hoc* comparisons indicated that the right frontal cortex was activated more than the left frontal cortex, and the bilateral supratemporal lobes were also activated more than the occipital and parietal lobes (*p*s < 0.05). These observations were consistent with previous findings that auditory MMNs tend to be generated from both the right frontal cortex and the bilateral supratemporal cortex (Näätänen et al., 2007; Saint-Amour et al., 2007). However, the activations of the Cantonese and Mandarin speakers did not exhibit significant differences for either cortex.

#### VO Condition

**FIGURE 3:**
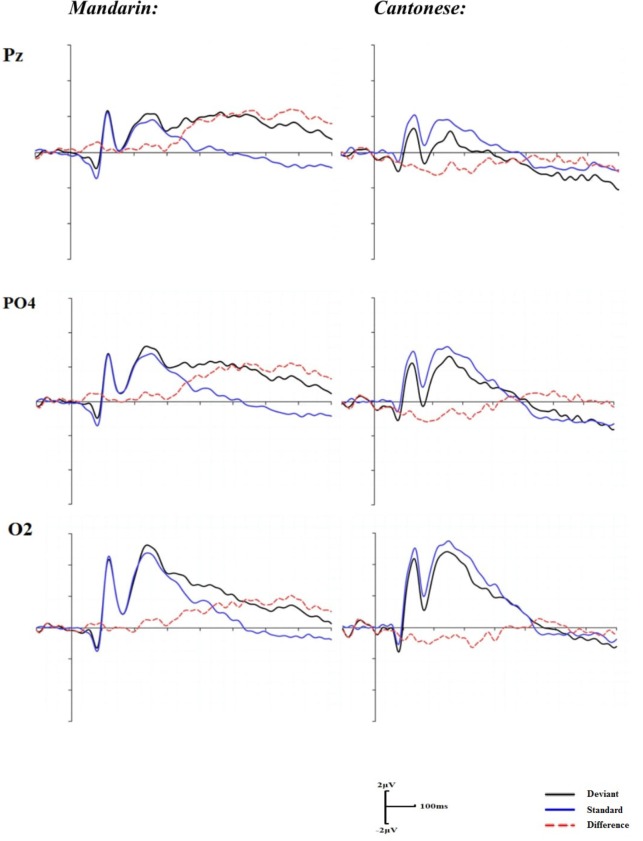
Grand mean ERPs for the standard, deviant trials, and difference waveforms in the visual condition.

Similar to the AO condition, repeated 2 (groups) × 16 (electrodes) two-way ANOVAs were performed in the time window of 180–320 ms after articulation. The main effect of group was non-significant, *F*(1,22) = 1.72, *p* = 0.203*;* however, a significant main effect of electrodes was identified, *F*(15, 30) = 4.36, *p <* 0.05, η^2^ = 0.17. A significant interaction of groups by electrodes was also revealed, *F*(15,330) = 2.60, *p <* 0.05, η^2^ = 0.12. The *post hoc* comparisons indicated that the mean amplitudes of the difference waveforms between Cantonese and Mandarin were significantly different at Pz, PO4, and O2 (*p*s < 0.05) (**Figure 3**).

#### AV Condition

**FIGURE 4:**
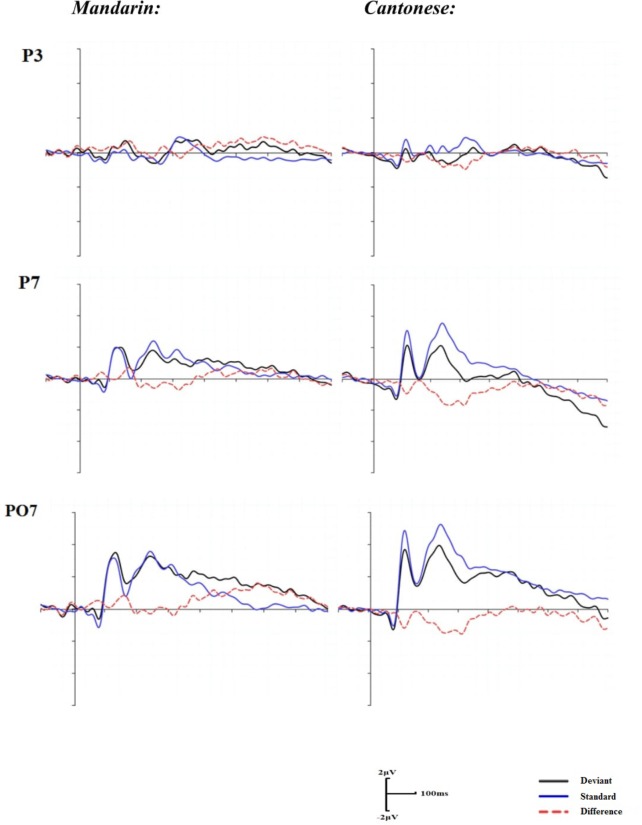
Grand mean ERPs for the standard, deviant trials and difference waveforms in the audiovisual condition.

With groups as a between-subject factor and electrodes as a within-subject factor, repeated two-way ANOVA was conducted in the time window of 160–280 ms after articulation. There was no significant main effect of groups, *F*(1,22) = 1.10, *p* = 0.31, although a significant main effect of electrodes was identified, *F*(15,330) = 3.00, *p <* 0.01, η^2^ = 0.13. A significant interaction of electrodes by groups was also demonstrated, *F*(15,330) = 2.40, *p <* 0.05, η^2^ = 0.11. The *post hoc* comparisons indicated significant group differences at the electrodes of P3, P7, and PO7 (**Figure 4**). The mean amplitudes of the difference waveforms at the three electrodes for the Cantonese speakers group were more negative than those for the Mandarin group (*p*s < 0.05).

To summarize, the results of Experiment 2 suggested that Cantonese speakers were more sensitive to the stimuli differences in both the visual and audiovisual conditions than the Mandarin speakers, although their sensitivity to the variations of auditory stimuli was equivalent.

## Discussion

Previous work has demonstrated that the McGurk effect generated by Chinese speakers was weaker than those of Japanese speakers (Sekiyama, 1997; Hayashi and Sekiyama, 1998). The authors attributed the weak illusions produced by Chinese participants to their tonal language background. According to their argument, Chinese speakers whose first language is tonal rely more on auditory cues because the information acquired from the auditory modality is more effective for them to perceive tonal information, and thus, visual cues are less utilized by them as compared to Japanese speakers. However, those studies were criticized for the confounding of the bilingual experience and cultural differences. More importantly, the weaker McGurk effect by Chinese speakers might also result from the simpler segmental phonology of Chinese and the consequent less utilization of visual information as compared to their Japanese counterparts. However, this possibility was not discussed in the study by Sekiyama (1997). In order to test the role of tonal and segmental features on the McGurk effect with the bilingual and cultural factors controlled, Mandarin and Cantonese speakers, who came from the same cultural background without any experiences of living abroad, were chosen as our participants in the present study.

Behavioral and ERP studies were conducted to compare the McGurk effects generated by the two groups and investigate the mechanism underlying the group difference. Experiment 1 revealed that Cantonese speakers had a stronger McGurk effect than the Mandarin speakers in audiovisual perception. However, no group difference was identified for either auditory or visual speech perception. In Experiment 2, the oddball paradigm was utilized and MMNs evoked under the auditory, visual, and audiovisual conditions were explored. The results revealed significant group differences in both the visual and audiovisual conditions although the two groups performed equally in the auditory condition. More specifically, the violation of articulation in the VO condition elicited a more negative MMN for Cantonese speakers than for Mandarin speakers in the right occipital cortex, which is associated with face processing as described in previous research (Pitcher et al., 2007). This finding illustrated that Cantonese speakers were more sensitive to the change of visual information than were Mandarin speakers. The sensitivity to the violation of visual stimuli could also elicit a more negative McGurk-MMN in the AV condition for the Cantonese group than for the Mandarin group, which echoes the behavioral finding that the Cantonese group exhibited a stronger McGurk effect than the Mandarin group. In addition, in the AV condition, the auditory cortex was activated even when the real acoustic change was absent, which was consistent with previous findings (Sams et al., 1991; Kreegipuu et al., 2013).

The current findings support our second prediction that Cantonese speakers rely more on visual cues due to the more complex segmental phonology of Cantonese and consequently exhibit a stronger McGurk effect as compared to Mandarin speakers. The results can be further explained under the framework of the Fuzzy Logic Model of Perception (FLMP). According to FLMP, in audiovisual speech perception, the complexity and ambiguity of auditory information will call for the compensation of the visual modality (Massaro, 1989). Compared to Mandarin, Cantonese is phonologically more complex in both tonal and segmental aspects, which result in the higher ambiguity in perceiving tones and segmental information in Cantonese than in Mandarin (Gao et al., 2000; Lee and Qian, 2007). However, the effectiveness of visual cues in perceiving tonal and segmental information is different. Lexical tones are produced by vibrating the vocal throat and different pitches are generated through the pressure on laryngeal muscles (Chen and Massaro, 2008). Thus, rigid head movements, rather than lip movements are associated with lexical tone perception. Furthermore, such visual cues are mainly useful for discriminating contour tones (Burnham et al., 2001, 2015). However, compared to the tonal aspect, visual cues for Cantonese segmental phonology are more visually identifiable because segmental phonology can be cued through lip movement and the place of articulation (Lansing and McConkie, 1999; Leung et al., 2004; Chen and Kent, 2010). Since the McGurk effect is not caused by the illusive integration of auditory sound and head movements, the discrepancy between Cantonese and Mandarin in the tonal aspect might not have much impact on the McGurk effect. However, visual cues such as lip movements which are important for perceiving segmental phonology are associated with the McGurk-illusion. Therefore, intra-linguistic difference in segmental phonology seems to influence the visual involvement and, consequently, the McGurk effect in audiovisual perception. As Cantonese speakers relied more on visual cues (e.g., lip movements, the place of articulation) to perceive segmental information, they demonstrated a stronger McGurk effect than Mandarin speakers.

In fact, the influence of segmental phonology on audiovisual speech perception has been reported in previous studies. In some other studies conducted by Sekiyama et al. (2003) and Sekiyama and Burnham (2008), it was found that Japanese speakers showed a weaker McGurk effect than their American counterparts because the segmental structure of Japanese is simpler than that of English and visual cues were more effective in identifying segmental phonology (e.g., more visually identified labials) in English than in Japanese. In another study, Sekiyama’s et al. (2003) group compared the McGurk effect between Japanese and English children and adults, respectively. The group difference in McGurk effect was only found among adults that adult English speakers showed stronger McGurk effect than their Japanese counterparts. However, English children and their Japanese counterparts performed equally on the McGurk effect. The increasing reliance on visual cues among English speakers was due to their incremental exposure to English, whose segmental phonology was complicated. While for Japanese speakers, the segmental structure of their native language was simpler than that of English and therefore the requirement of the supplement of visual information was not so high.

## Conclusion

The present study has revealed that Cantonese speakers relied more on the visual modality when perceiving audiovisual speech, and therefore exhibited a stronger McGurk effect than Mandarin speakers through behavioral and ERP measurements. The current findings suggest that segmental rather than tonal complexity may be more important in determining the McGurk effect generated by those speaking different Chinese dialects. Future studies are awaited to extend our findings to intra-linguistic comparisons on the McGurk effect in other tonal languages and to inter-linguistic comparisons between tonal and non-tonal languages.

## Author Contributions

JZ, YM, and ZY designed and conducted the experiments. YM performed the data analyses. JZ and YM took the lead in manuscript writing. ZY, CM, and XF made significant contributions to data interpretations and manuscript revision.

## Conflict of Interest Statement

The authors declare that the research was conducted in the absence of any commercial or financial relationships that could be construed as a potential conflict of interest.
